# Cardiovascular Imaging in Stress Cardiomyopathy (Takotsubo Syndrome)

**DOI:** 10.3389/fcvm.2021.799031

**Published:** 2022-01-28

**Authors:** Fawzi Zghyer, W. Savindu Pasan Botheju, Joshua E. Kiss, Erin D. Michos, Mary C. Corretti, Monica Mukherjee, Allison G. Hays

**Affiliations:** ^1^Division of Cardiology, School of Medicine, Johns Hopkins University, Baltimore, MD, United States; ^2^Virginia Commonwealth University Medical Center, Richmond, VA, United States

**Keywords:** cardiomyopathy, CMR (cardiovascular magnetic resonance), echocardiography, stress cardiomyopathy, imaging

## Abstract

Stress cardiomyopathy (Takotsubo syndrome) is a reversible syndrome stemming from myocardial injury leading to systolic dysfunction and is usually noted in the setting of a stressful event, be it an emotional or physical trigger. While the exact pathophysiology behind stress cardiomyopathy is yet unknown, there is ample evidence suggesting that neurocardiogenic mechanisms may play an important role. Although historically stress cardiomyopathy was generally thought to be a relatively benign condition, there is growing recognition of the cardiovascular complications associated with it despite its reversibility. Our review aims to shed light onto key cardiovascular imaging modalities used to diagnose stress cardiomyopathy while highlighting the role that imaging plays in assessing disease severity, identifying complications, dictating treatment approaches, and in short-term and long-term prognosis.

## Background

Takotsubo syndrome, also known as stress cardiomyopathy (stress CM), apical ballooning syndrome, or broken heart syndrome, is increasingly recognized as an important cause of acute reversible myocardial injury and acute systolic dysfunction (1). The origin of the name “takotsubo” refers to octopus traps used in Japan, which resemble the apical ballooning pattern often observed on fluoroscopic left ventriculogram in the classic presentation of stress CM (2). Previously thought to be a rare diagnosis, stress CM currently comprises around 2% of all the patients presenting with concern for acute coronary syndrome (ACS) (3).

Stress CM was initially thought to be a relatively benign, reversible condition, but as prevalence has increased it has become apparent that it is often associated with significant morbidity and mortality (1). There is increasing recognition of associated cardiovascular complications such as left ventricular (LV) outflow tract obstruction, mitral regurgitation, and heart failure which may all lead to cardiogenic shock (4).

In an era where the application of multimodality cardiovascular imaging has been increasing, our review will aim to highlight the diagnosis of stress CM with a particular focus on imaging.

## Definition and Diagnosis

Stress CM is defined as a syndrome of transient and acute LV systolic and diastolic dysfunction usually related to a profound emotional or physical stress within the prior 5 days (1, 5). The syndrome is suspected when observed regional wall motion abnormalities (RWMA) cannot be explained by corresponding coronary artery occlusions. The typical pattern of LV RWMA that is described includes apical hypokinesia, akinesia, or dyskinesia which gives the apical ballooning shape with relative basal hyperkinesis. Other forms of stress CM include “reverse Takotsubo” or “atypical Takotsubo” which are characterized by mid-ventricular or basal hypokinesis (6). Focal stress CM is another rare form described by its focal LV RWMA which makes it very hard to distinguish from myocarditis or myocardial infarction (7). Clinically, stress CM often presents with ST-segment changes on electrocardiography (ECG), elevation in troponins, a significant increase in serum natriuretic peptides, and characteristic imaging findings. However, coronary angiography remains an essential diagnostic modality to definitively exclude ischemic etiologies for RWMA (1).

Multiple diagnostic criteria have been proposed: the most commonly used are the Heart Failure Association of the European Society of Cardiology diagnostic criteria for Takotsubo Syndrome (8), the International Takotsubo Diagnostic Criteria (InterTAK) (9), and the Revised Mayo Clinic Criteria (10). The criteria are summarized in Table 1. The Japanese Society of Echocardiography (JSE) and the European Association of Cardiovascular Imaging (EACVI) published a joint consensus document in 2020 providing a comprehensive review of the various cardiovascular imaging modalities that can be utilized in diagnosing and prognosticating stress CM. They incorporated the InterTAK criteria into a simplified diagnostic algorithm that helps evaluate chest pain and/or dyspnea and rule out several cardiac causes before getting to stress CM (11).

**Table 1:** The table below summarizes the diagnostic criteria for stress cardiomyopathy.

**Heart Failure Association-European Society of Cardiology Criteria** • Transient RWMA in LV or RV which are frequently but not always accompanied by a stressor • The RWMA usually extends beyond a single coronary artery with circumferential involvement of the segments involved • The absence of acute coronary syndrome • New and reversible electrocardiographic abnormalities • Significantly elevated serum natriuretic peptides • Mildly elevated cardiac enzymes • Recovery of LV systolic function on cardiac imaging at follow-up (3–6 months)
**International Takotsubo Diagnostic Criteria (InterTAK Diagnostic Criteria)** • Transient LV dysfunction presenting with apical ballooning or mid-ventricular, basal, or focal wall motion abnormalities. RV involvement can be present too. The regional wall motion abnormalities usually extend beyond a territory of a single coronary artery • An emotional physical or combined trigger precedes the symptoms, however not obligatory • Neurologic disorders as well as pheochromocytoma may serve as triggers for stress cardiomyopathy • New ECG abnormalities are present • Cardiac biomarkers are moderately elevated, but natriuretic peptides are significantly elevated • Significant coronary artery disease is not a contradiction in Takotsubo syndrome • Patients have no evidence of infectious myocarditis • Postmenopausal women are predominantly affected
**Revised Mayo Clinic Criteria** • Transient hypokinesis, akinesis, or dyskinesis of the LV with or without apical involvement, the RWMA extends beyond a single coronary artery distribution; a stressful trigger is often but not always present • Absence of obstructive coronary artery disease or angiographic evidence of a plaque rupture • New ECG abnormalities or moderate elevation in cardiac biomarkers • Absence of pheochromocytoma or myocarditis

## Pathophysiology

The exact pathophysiology leading to stress CM still remains unknown. There is a known neuro-cardiovascular link that may contribute to stress CM, but recently neuroimaging studies have given us more insight (12). The central autonomic nervous system (CANS) is vital for cardiovascular regulation. Prior to adopting the terms Takotsubo and stress CM, clinicians have identified instances of transient systolic dysfunction as well as dynamic EKG changes in relation to intracranial pathology such as stroke or hemorrhages (12). This phenomenon has been later described as “neurogenic stunning” and was also seen in patients undergoing electroconvulsive therapy (ECT) (13). As described by de Chazal et al. in a recent review, in the acute phase, there is increased blood flow in the hippocampus, brainstem, and basal ganglia with return to normal upon resolution of the syndrome (1). However, it is still unknown why some emotional stressors cause myocardial dysfunction while other stressors do not. Stress induces activation of complex neocortical limbic pathways which leads to the activation of brainstem noradrenergic neurons and stress related neuropeptides (NPY) produced by the arcuate nucleus of the hypothalamus. Acute stressors induce brain activation, which increases the bioavailability of norepinephrine, cortisol, and NPY. Both circulating epinephrine and norepinephrine released from the medulla of the adrenal glands or locally from sympathetic nerve terminals are known to be significantly increased during the acute phase of stress CM. The catecholamine and NPY surge can lead to the apical ballooning physiology through multiple mechanisms including direct catecholamine toxicity, epicardial, and microvascular coronary spasm or vasoconstriction, and increased cardiac afterload (14). Experimental data have shown that β_2_-adrenoceptors are more frequently expressed in an apical than the basal segment of the LV, while a reverse distribution is present for norepinephrine β_1_-adrenoceptors and sympathetic and sympathetic nerve terminals of the neuro-cardiac axis, which are dense at the base but not the apex (15). Previously, it was widely thought that the catecholamine surge is solely responsible for the cardiomyopathy; however, it is increasingly recognized that alternate mechanisms may play more of a role. Catecholamine levels often increase in response to cardiac dysfunction and hypotension. It is not yet clear how norepinephrine and NPY contribute mechanistically to stress CM. One hypothesis maintains that the cardio-depressant effects of catecholamines may lead to supply demand mismatch at the level of the myocardium and eventually myocardial stunning (1). Thus, it is plausible that in individuals with higher levels of NPY/norepinephrine, an intense stressor may predispose them to develop stress CM (1).

## Risk Factors

Estrogen deficiency in post-menopausal women appears to be one of the strongest risk factors associated with stress CM. This is often superimposed on a background of anxiety or panic disorder (16). Diabetes has also been described as a risk factor, as 10–25% of all the patients presenting with stress CM have concomitant diabetes (17). One of the non-cardiac risk factors frequently associated with stress CM is asthma, particularly after the use of medical interventions such as beta-2 agonists, racemic epinephrine, and intubation. It is not fully understood whether it is the asthma itself or the treatment that triggers the syndrome (18). Marijuana use is also associated with stress CM with a subsequent higher mortality (19). Prior studies suggest that cannabinoids induce NPY expression from the arcuate nucleus of the hypothalamus which can lead to neurogenic myocardial stunning (20). Finally, as we continue to assimilate further data on the COVID-19 pandemic, there is increasing evidence of acute or chronic cardiac injury in the setting of a COVID-19 infection. Although stress CM has been reported in the setting of COVID-19, other pathologies as a cause of cardiac injury are more common including supply/demand mismatch ischemia, microvascular thromboses, myocarditis, or cytokine storm from a hyperinflammatory state (21–24).

## Echocardiographic Findings

### Left Ventricular Wall Motion and Systolic Function

**Figure 1:**
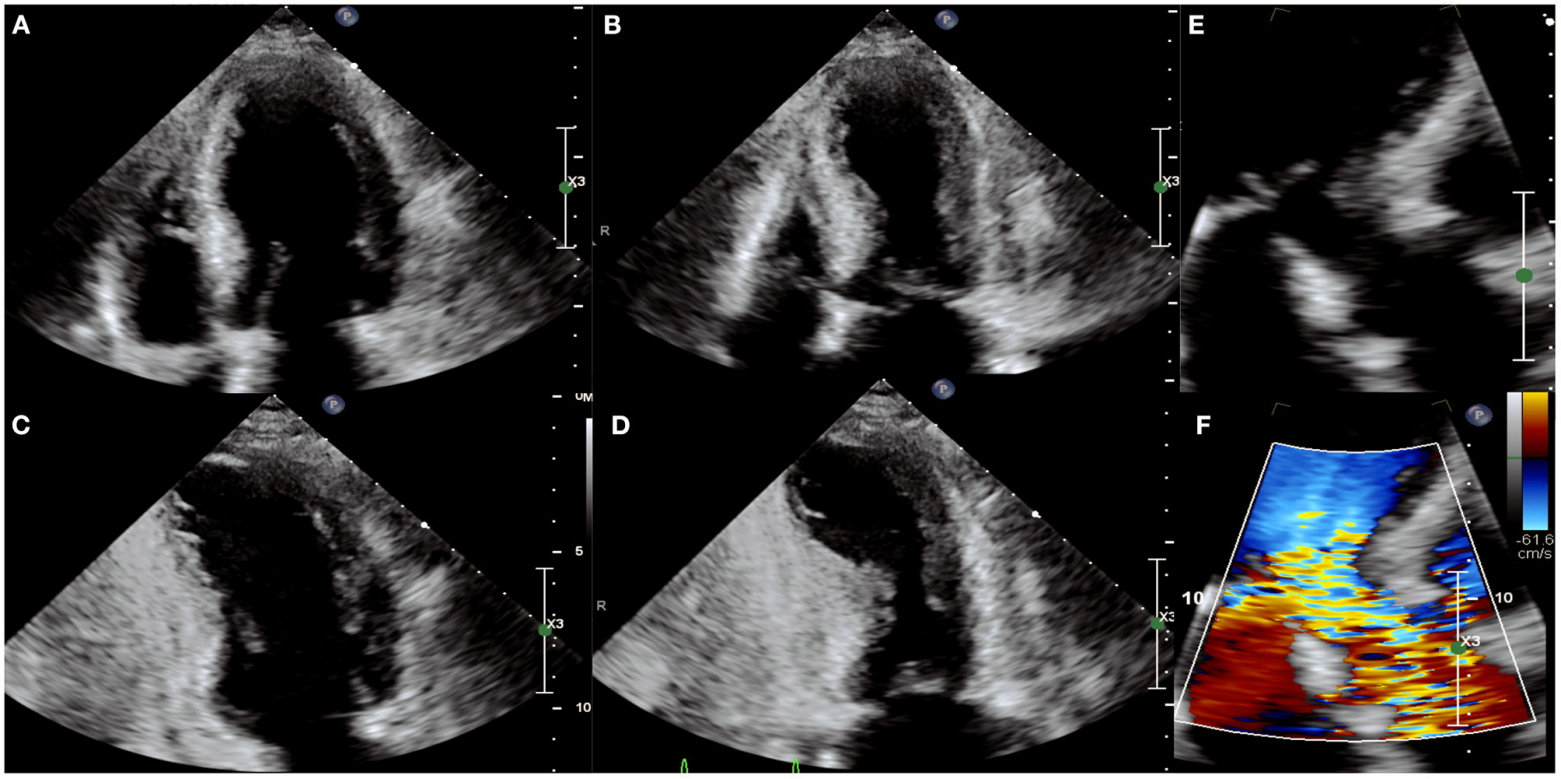
Echocardiographic images of a person with stress cardiomyopathy with left ventricular outflow track obstruction (LVOT) and systolic anterior motion (SAM) of the mitral valve. The apical-4-chamber view in diastole **(A)** and systole **(B)** and 2-chamber view in diastole **(C)** and systole **(D)** showing classic apical ballooning with akinesis and hyperdynamic basal segments. **(E,F)** Illustrate SAM and turbulence across the LVOT indicative of LVOT obstruction.

In the acute phase of stress CM, an echocardiographic assessment is an essential first step toward the diagnosis. The typical echocardiographic findings are characterized by the presence of a large area of dysfunctional myocardium which extends beyond one coronary artery territory. In classic stress CM, symmetrical wall motion abnormalities are seen with akinesis or dyskinesis of the apical and mid-ventricular segments of the anterior, lateral, septal, and inferior walls with hyperdynamic function of the basal segments (25) (Figure 1). Other variants such as “mid-ventricular” or “inverted” stress cardiomyopathy can also be seen. The mid-ventricular variant is characterized by akinesis of the mid-ventricular segments with mild hypokinesis or normal contraction of the apical segments and hypercontractility of the base. The inverted variant has two different forms: the first manifests as preserved apical function and severe hypokinesis of the rest of the walls while the second form is characterized by hypokinesis only in the basal segments (often called “reverse Takotsubo”) (25).

Echocardiography also demonstrates reduced LV ejection fraction (EF) with variable severity and in most cases recovers as the myocardial stunning resolves (2). The degree of the EF reduction varies depending on the level of myocardial impairment which can often correspond with pre-existing comorbidities, sex, and age. While the degree of myocardial impairment can sometimes be severe, this is not typically reflected by the degree of elevation of cardiac biomarkers, which may be only modestly elevated. The discrepancy between troponin levels and RWMAs can help clinicians to differentiate between ACS and stress CM. More recently, the product of Troponin I level and echocardiographic LVEF has been utilized as an index to differentiate stress CM from ST elevation myocardial infarction (STEMI). A value ≥250 is indicative of a STEMI, with a sensitivity and specificity of 95 and 87%, respectively (26).

Left ventricular EF has been found to be an independent predictor of major cardiovascular complications and can help identify stress CM patients at higher risk particularly in those aged >75 years (27). Elderly patients demonstrate significantly lower LV systolic function compared to younger patients (defined as <75 years) (25). If RWMAs persist, then it is reasonable to evaluate with a cardiac MRI to rule out necrosis or other pathologies.

Assessment of LV longitudinal strain may have an important prognostic value in the acute phases of stress CM. Decreased global longitudinal strain (GLS) in patients with stress CM was found to correlate with higher in-hospital mortality (28).

### LV Diastolic Function

LV diastolic dysfunction is common in stress CM (29). Global diastolic dysfunction has been observed in some patients in the early phases of stress CM which is evident by impaired left ventricular relaxation and increased E/e' ratio, a non-invasive marker of LV filling pressures (30, 31). In the Takotsubo Italian Network (TIN) registry, E/e' ratios were higher in patients who went on to develop acute decompensated heart failure and was found to be an independent predictor of in-hospital mortality (32). Of all the complications associated with stress CM, acute decompensated heart failure is the most common early complication; thus, an early assessment of the E/e' ratio may allow for early identification of patients at higher risk of decompensation and could serve as a useful tool to guide management. As E/e' ratio elevations can be transient, the ratio itself could be used as a marker for functional recovery when followed serially across the course of the syndrome.

### Mitral Regurgitation (MR)

The mechanism of MR in stress CM is not completely understood. Systolic anterior motion of the anterior mitral leaflet (SAM) has been reported in 40–50% of patients with stress CM and may be associated with significant MR (33) (Figure 1). MR in stress CM is typically described in cases where reduction in LVEF is severe and LV volume is high and likely represents functional MR due to papillary muscle displacement and tethering of the mitral leaflets in the setting of a dilated and dysfunctional LV (33). In the TIN registry, the presence of MR appeared to be a powerful prognostic marker associated with cardiogenic shock and in-hospital morbidity and mortality (32).

### LV Outflow Tract Obstruction (LVOTO)

LVOTO, diagnosed by echocardiogram, may result from basal hypercontractility with or without systolic anterior motion of the anterior mitral leaflet causing dynamic obstruction of the LV outflow tract (LVOT), and is seen in as many as 12.5–25% of cases (32, 34). Significant LVOTO is described as a peak instantaneous LVOT gradient ≥ 30 mmHg. The LVOT gradient has important clinical and therapeutic implications in patients with stress CM; in this cohort, inotropic agents and diuretics should be avoided as the basal hypercontractility induced by catecholamines and the reduction in preload can lead to an exacerbation in gradient with subsequent hemodynamic collapse and cardiogenic shock. LVOTO is associated with increased LV afterload and systolic wall tension which may lead to subendocardial ischemia. In this setting, beta blockade may be beneficial in reducing inotropy and reduced myocardial demand. Alpha-1 agonism with phenylephrine can increase systemic vascular resistance and thus help reduce the effect of dynamic LVOT obstruction, ultimately reducing LV afterload and wall stress (25). Patients with severe LVOTO and stress CM are at higher risk for free wall rupture and life-threatening arrhythmias.

### Right Ventricular (RV) Function

Electrocardiographic signs of RV strain warrant special attention with regards to evaluation of RV function in stress CM.

The prevalence of RV involvement has been reported to be around 14% in one study; however, this may be an underestimation due to difficulty imaging the RV (25). In patients with biventricular ballooning, RV contraction often mirrors that of the LV (35). The pattern described is the opposite of the well-described McConnell's sign seen in massive pulmonary emboli which manifests itself as RV apical hyperkinesis and basal and mid hypokinesis to akinesis. The pattern of RV involvement was eventually called “reverse McConnell's sign” (36). There is conflicting evidence on whether RV involvement predicts worse outcomes. In the TIN registry, RV involvement was more prevalent in patients with major complications. No significant correlation has been found between tricuspid annular systolic excursion (TAPSE) and major adverse events (32).

### Contrast Echocardiography

Per the updated American Society of Echocardiography guidelines (2018) and European Association of Cardiovascular Imaging (2017) guidelines, contrast echocardiography is a recommended tool to evaluate for the presence of an LV thrombus in situations where it is not detected on non-contrast echocardiography (37). However, if contrast echocardiography fails to detect a thrombus in cases with a high clinical suspicion for a thrombus, cardiac MRI would provide a higher sensitivity and specificity at detecting LV thrombi.

## Cardiac Magnetic Resonance Imaging (CMR)

### Cine CMR Sequence

**Figure 2:**
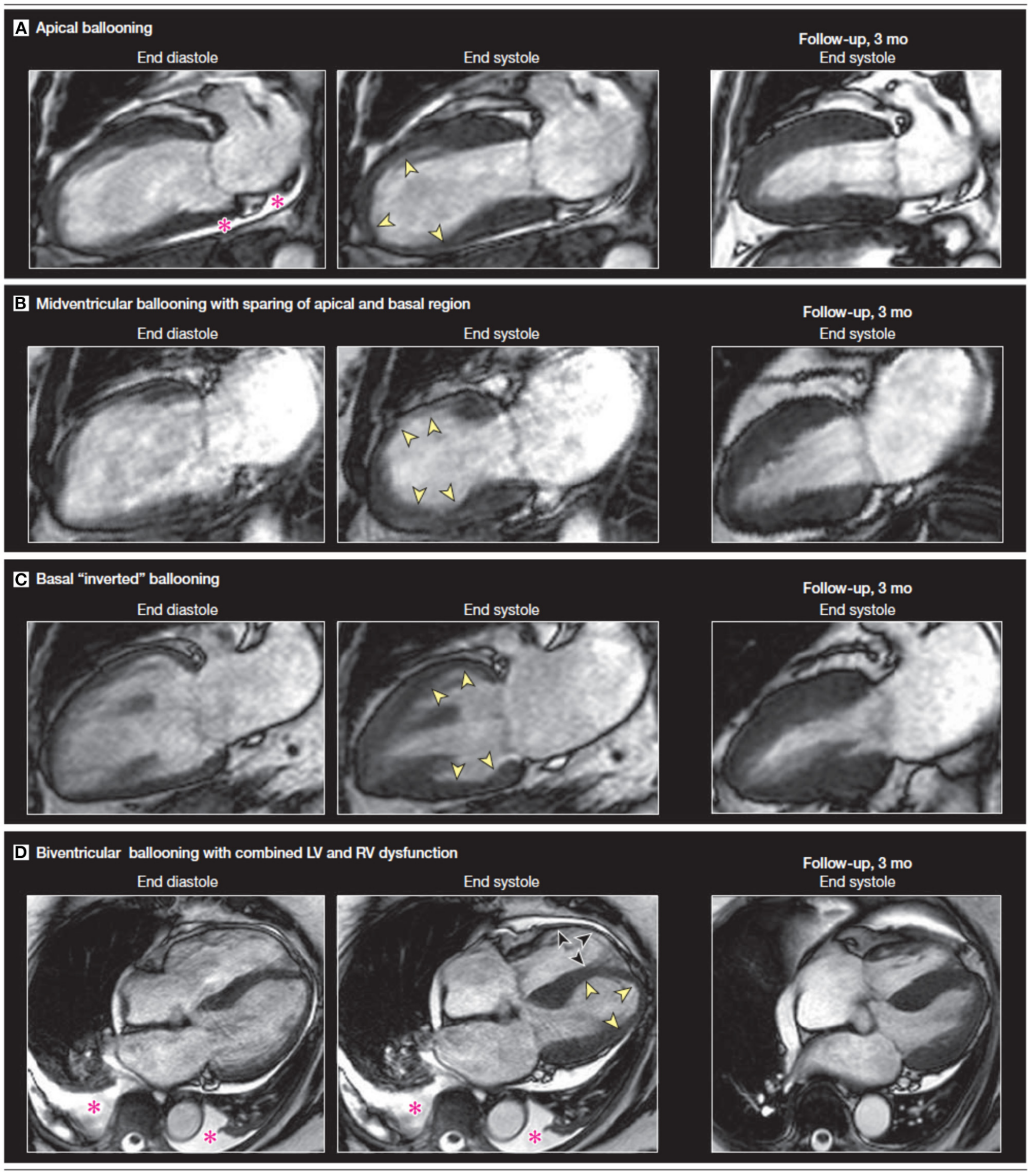
A comparison of vertical long axis CMR cine sequence images on diagnosis and at 3-month follow-up showing a recovery of wall motion abnormalities. The four sets of images display four different variants associated with stress CM. The pink asterisk denotes a pericardial effusion. Yellow arrows indicate apical akinesis while the black arrows indicate RV apical akinesis in the biventricular ballooning variant. Figure adapted from Figure 2 on Clinical Characteristics and cardiovascular magnetic resonance findings in stress (Takotsubo) cardiomyopathy by Eitel et al. (41).

Cine CMR sequences allow for more detailed assessment of LV and RV function, ventricular wall motion abnormalities, and possible complications of stress CM (38, 39). While the “apical ballooning” pattern of wall motion accounts for around 75–80% of patients, stress CM can also present in the form of other less common variants, namely the mid-ventricular variant or the inverted variant which are discussed earlier (40, 41) (Figure 2). The cine sequence can also be used to demonstrate LVOTO with or without systolic anterior motion of the mitral valve leaflet as well as functional mitral regurgitation (39). These complications can be further assessed with phase contrast velocity imaging in order to generate quantitative values such as LV outflow tract gradient.

Additionally, CMR allows for more quantitative assessment of RV function. RV involvement has been assessed by CMR in multiple studies: patients with RV dysfunction were older, had with longer hospital stays, had more frequent preceding stressful events, had significantly more pleural effusions, and had a lower LV EF compared to those that did not have RV involvement (41, 42).

Recently, CMR post-processing techniques called feature/tissue tracking CMR (FT-CMR) have been developed that enable strain analysis for the quantification of myocardial deformation. A study by Stiermaier et al. evaluated 141 patients with stress CM and found that reduced global circumferential (GCS) and global longitudinal strain (GLS) were associated with the apical ballooning variant while reduced global radial strain (GRS) was associated with the basal ballooning variant (43). Additionally, the study posited that GLS may be useful as a prognostic indicator as LV strain measures worse than −14.75% were associated with adverse outcomes (43).

### Myocardial Edema

**Figure 3:**
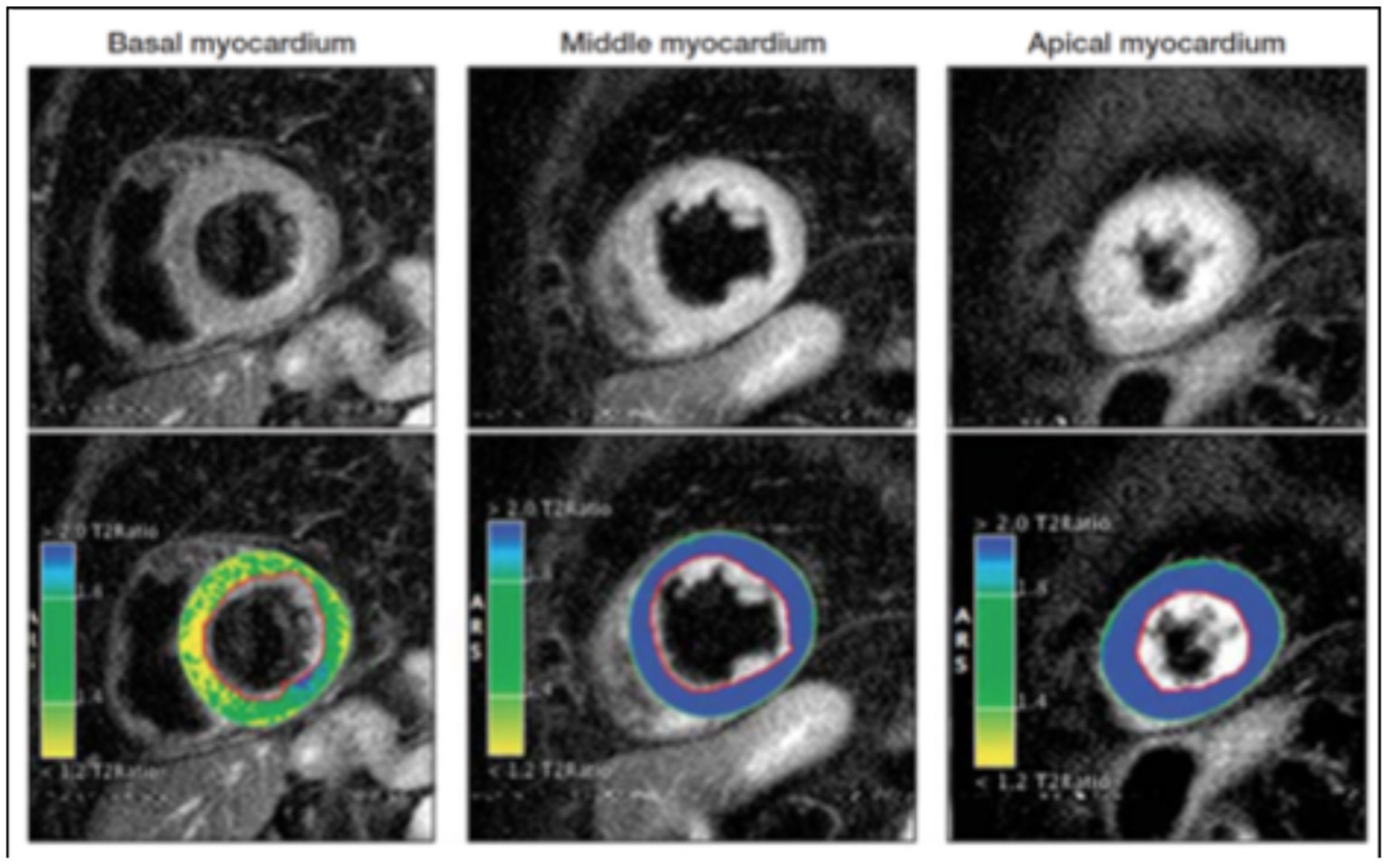
T2-weighted sequences demonstrating variability in signal intensity based on the affected myocardial regions. The color coding resembles varying signal intensity ratios between myocardium and skeletal tissue with blue indicating a ratio ≥ 1.9 (indicating edema) while yellow/green indicates a ratio <1.9, indicative of normal myocardium. These images illustrate predominant myocardial edema in the mid-ventricular and apical segments. Adapted from Clinical Characteristics and cardiovascular magnetic resonance findings in stress (Takotsubo) cardiomyopathy by Eitel et al. (41).

In the setting of myocardial injury, tissue inflammation may increase resulting in localized myocardial edema (41). Changes in the tissue water content can be evaluated via a CMR T2-weighted imaging protocol (Figure 3) which allows for assessment of the distribution of myocardial edema and can be helpful in differentiating between cardiac pathologies such as stress CM, ACS, and myocarditis.

By employing the black blood T2-weighted (fast spin echo) triple-inversion recovery (IR) sequence, fat, and flowing blood can be suppressed allowing the contrast signal to differentiate between normal myocardium and edematous tissue (44). This process can be quantified by calculating the ratio of the signal intensity (SI) between the myocardium and skeletal tissue, with a cut-off value of over 1.9 considered significant for edema.

The pattern of edema in stress CM classically overlaps with the dysfunctional ventricular regions noted on the cine CMR sequence and does not correspond to territories served by epicardial vessels that would generally be seen in ACS (41). The signal distribution tends to be transmural and circumferential over the affected area, unlike in myocarditis where it tends to be more diffuse and heterogenous with a propensity to have higher signal intensity in the sub-epicardial or mid-myocardial tissue.

It is important to note, however, that T2-weighted triple IR can be prone to artifacts from various factors such as arrhythmias, blood pooling, or breathing motion artifact. Therefore, studies investigating techniques that use motion correction algorithms such as extracellular volume (ECV) mapping and parametric T1 or T2 mapping have shown to improve diagnostic accuracy and provide further quantitative evidence in evaluating myocardial edema (15, 45–47). In patients with myocardial infarction, the intensity of T2 signaling tends to take months or even years to decline in the infarcted and surrounding tissue depending on the severity of the injury. In stress cardiomyopathy, there has been variable data with some studies showing a normalization of signaling as the myocardial function returns to normal, while other studies have shown a slower return to baseline, despite recovery in LV myocardial function (15, 45–48). These novel techniques could prove to be viable alternatives to evaluate myocardial inflammation and characterize disease severity without using gadolinium contrast and warrant further studies with larger patient cohorts.

Although the pathophysiology of myocardial inflammation in stress CM is not well-known, increased myocardial edema on imaging may indicate a direct inflammatory process from the syndrome, a secondary effect stemming from elevated sympathetic drive, or microvascular ischemia associated with stress CM (41, 48, 49).

### Late Gadolinium Enhancement (LGE)

The use of gadolinium-mediated myocardial enhancement has been used to delineate areas of myocardial scarring or replacement fibrosis stemming from myocardial injury and inflammation. Conventionally, when using LGE to delineate areas of fibrosis in patients with a history of myocardial infarction or myocarditis, a threshold of 5 standard deviations (SDs) above the signal intensity seen in remote, unaffected tissue is used to define significant enhancement. In areas affected by myocardial infarction, the LGE pattern is generally predominant in the subendocardial tissue and can spread transmurally depending on the extent of injury, while in myocarditis the distribution tends to dominate in the sub-epicardial region (updated Lake Louise consensus criteria) (50). Previously, it was thought that the absence of LGE was a pathognomonic feature of stress CM that differentiated it from other pathologies such as myocardial infarctions or myocarditis; however, several studies have now shown that stress CM does present with a certain degree of LGE that is usually of reduced intensity compared to other conditions that cause fibrosis (41, 51, 52). Eitel et al. showed no evidence of LGE in any of the 239 patients assessed in one study, while lowering the threshold to 3 SD showed positive findings in 22 patients (9%) (41). These patients had higher troponin levels at presentation compared to those that had negative LGE but there were no differences in the LV ejection fraction, end-diastolic volume, or end-systolic volume. Another study similarly revealed that changing the SD threshold for LGE may yield different results (53).

There are various theories as to what causes the delayed enhancement, with some studies suggesting a delayed washout of contrast due to the presence of myocardial inflammation and interstitial edema. Other studies have used immune-histological evidence to show that affected areas in stress CM have increased collagen-1 which could be indicative of a fibrotic process (52, 53). Perhaps the low level of LGE reflects the reversibility of stress CM and near or complete resolution, which is an essential diagnostic component of this condition. In ischemic cardiomyopathies or non-ischemic cardiomyopathies such as sarcoidosis, higher degrees of LGE are often associated with poorer prognoses due to irreversible myocardial injury (54). In one study, stress CM patients with LGE had a higher frequency of cardiogenic shock and had a slower recovery of wall motion compared to those that did not display LGE (53). Therefore, extent of LGE on CMR may represent a useful prognostic marker in stress CM.

### Early Gadolinium Enhancement (EGE)

Within 1–3 min of gadolinium administration, EGE can be accessed via a high T1 inversion time. This technique is essential for the identification of LV thrombus. Thrombi are characterized by having no gadolinium uptake and are seen as a hypodense mass (almost black) in contrast to the gray myocardium. Important CMR parameters are summarized in Table 2.

**Table 2:** Important CMR parameters for the evaluation of stress CM.

**Imaging sequence**	**Clinical significance**
Cine CMR (balanced Steady State Free Precession/SSFP)	Assessing wall motion abnormalities
Feature/Tissue tracking CMR (FT-CMR)	Quantifying regional and global strain patterns
T2-weighted triple inversion recovery	Identifying areas with myocardial edema and distribution pattern
T1 or T2 mapping	Quantitative assessment of myocardial edema
First pass perfusion	Assessing perfusion defects
Early gadolinium enhancement	Ruling out LV thrombus
Late gadolinium enhancement	Assessing extent of regional inflammation and fibrosis

## Nuclear Imaging

Recent studies have reported a role for nuclear imaging findings in the diagnosis of stress CM. Single photon emission computed tomography can assess cardiac stunning using 201 Thallium or 99m Technetium-labeled radiopharmaceuticals and 123I-metalodobenzyl-guanidine (I-123 MIBG) (55). In addition, 18F-fluorodeoxyglucose (FDG) positron emission tomography can be used to detect myocardial glucose metabolism (56). In the acute and sub-acute phases of stress CM, the affected segments of the myocardium show defects in FDG and MIBG despite normal to slightly reduced perfusion (57). MIBG is a norepinephrine analog and uses the same mechanism of norepinephrine in the uptake and storage in presynaptic vesicles. After adrenergic stimulation, MIBG is released in the synaptic cleft but is not metabolized by monoamine oxidase (MOA) and catechol-O-methyltransferase (COMT) enzymes (58). MIBG is also labeled with iodine-123 (123I) as a radiotracer due to its suitability for imaging. With such properties, it is possible to evaluate the cardiac uptake of the MIBG and its distribution (59). Visually and quantitively, two parameters are analyzed: heart to mediastinal ratio (H/MR) and myocardial washout rate (WR) (59).

The evaluation of stress CM by I-123 MIBG scintigraphy demonstrates a defect in MIBG uptake at the level of the apex. Semi-quantitative measures also demonstrate a reduction in H/MR and an increase in washout (59). The use of nuclear imaging for the diagnosis of stress CM is promising but given the presence of alternative more accessible modalities, it is not the first line imaging modality for diagnosis.

## Cardiac Multi-Detector Computed Tomography

Cardiac multi-detector computed tomography (MDCT) is a comprehensive imaging modality that is used to assess cardiac function as well as coronary artery lesions (55). MDCT can provide valuable information regarding RWMA and can immediately rule out a coronary artery lesion with a high negative predictive value (60). In clinical practice, excluding the possibility of coronary obstruction or acute plaque rupture with MDCT still remains a challenge (55). Cardiac MDCT may be a promising imaging modality in the evaluation of suspected stress-induced cardiomyopathy. Cardiac MDCT may in particular be an appealing imaging modality when there is a clinical need to evaluate the coronary arteries at the same time or when there is a contraindication to MRI.

## Complications

**Figure 4:**
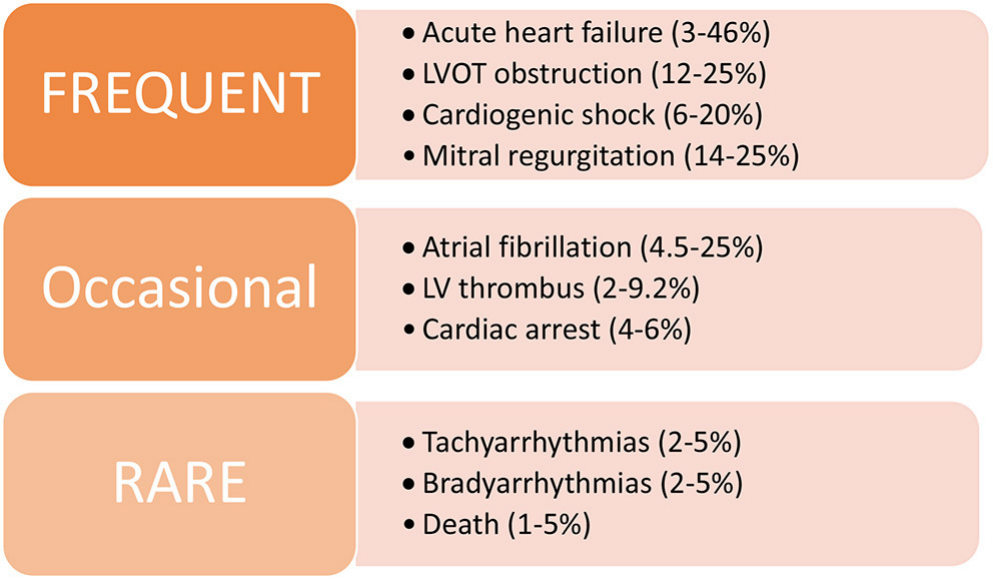
Summary of common in-hospital complications associated with stress CM.

Although the hallmark of stress CM is that myocardial function returns to normal within weeks, many patients endure complications related directly to stress CM or comorbid medical conditions and can have significant morbidity and mortality in the inpatient setting (Figure 4).

### Acute Heart Failure

Systolic heart failure is the most common complication encountered by these patients in the acute setting, with an incidence ranging between 3 and 46% (3, 32, 61, 62). This can occur as a result of depressed LV systolic function or secondary to other associated complications such as LVOTO or functional mitral regurgitation. Some patients have progression of heart failure to cardiogenic shock requiring inotropic or vasopressor support or even mechanical support via intra-aortic balloon pumps (IABP) or extracorporeal membrane oxygenation (ECMO), with a very small proportion of cases resulting in death. Biventricular involvement has a tendency to occur in the elderly population, is associated with lower LV ejection fraction, and is an independent predictor of adverse cardiovascular outcomes (41, 63). These patients tend to have longer and complicated hospital stays and have shown to have higher rates of in-hospital and long-term morbidity and mortality (64).

### LV Thrombus and Systemic Emboli

Formation of an LV thrombus is a relatively rare but significant complication associated with stress CM, particularly with the apical ballooning variant. Studies have shown an incidence between 2 and 9.2% of patients (65–68). In these cases, likelihood of clot formation is increased due to blood stasis from apical hypokinesis/akinesis increased stasis; furthermore, the adrenergic surge that is often thought to be a contributing mechanism behind stress CM could potentially cause endocardial damage that may initiate a thrombotic process. Stress CM patients with LV thrombus are at increased risk for systemic emboli including cerebrovascular embolic events, which can occur in around 17–25% of cases (66, 68).

### Arrhythmias

As cardiac arrhythmias are important potential complications of stress CM that could arise in the acute setting, patients need to be monitored closely via telemetry and serial ECGs in order to evaluate for abnormal rhythms and a prolonged QT interval. Atrial fibrillation can occur in ~4.5–25% of patients and is the most common arrhythmia seen in stress CM patients (69–71). Rarely, patients may develop more dangerous cardiac arrhythmias such as ventricular tachycardia or torsades de pointes, especially if a prolonged QT interval >500 ms is present (72, 73). In one study, men were more likely to have cardiac arrhythmias and a higher incidence of cardiac arrest compared to women (69).

## Treatment

### Acute Heart Failure and Cardiogenic Shock

**Figure 5:**
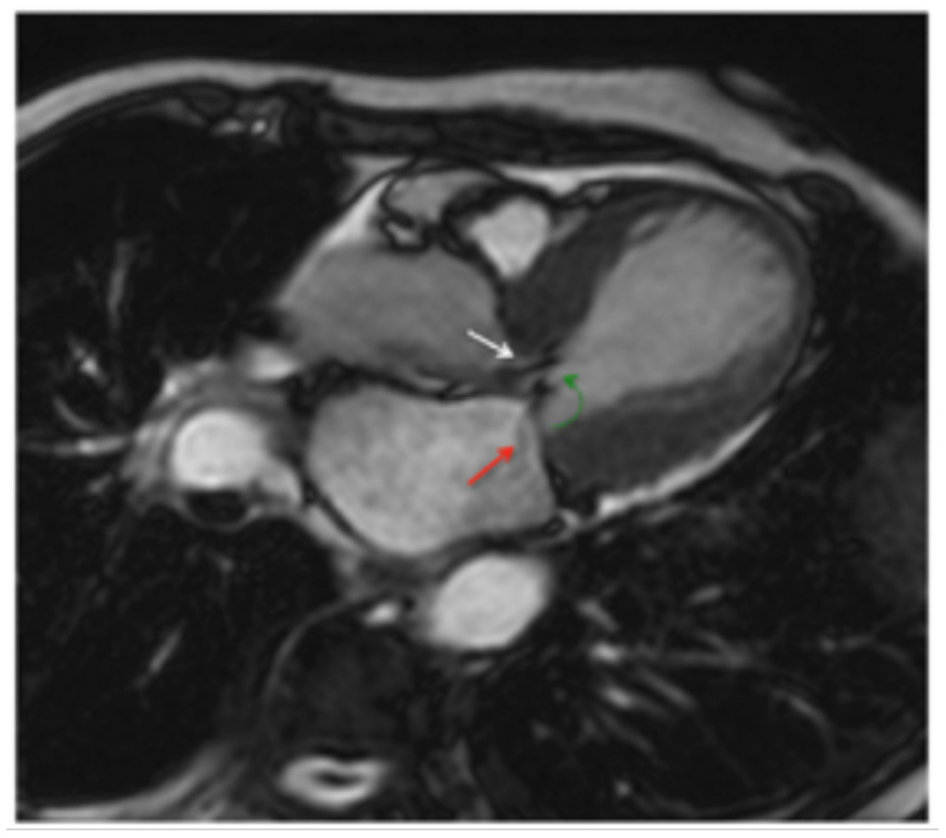
Cine CMR horizontal long-axis view illustrating the systolic “jet” seen in LVOT (white arrow) with concurrent systolic anterior motion of the mitral leaflets (green arrow) and functional mitral regurgitation (red arrow). Adapted from Figure 6 in Plácido et al. (39).

To date, there have been no large randomized controlled trials investigating various therapies that are commonly used for the treatment of acute heart failure in stress CM. The primary goal of therapy is to address any acute complications that arise, prophylactically treat to minimize risks of certain complications such as thromboembolism, and closely follow the patient throughout the course of the disease until full recovery and/or normalization of cardiac function. In determining the course of therapy, it is important to delineate the pattern of cardiomyopathy and identify certain features such as the presence of LVOTO that may require adjustment in medication regimen (Figure 5).

In a patient with normal cardiac output, the standard of treatment involves use of diuretics in order to achieve euvolemia and afterload reduction with arterial vasodilators (e.g., ACE-inhibiters, angiotensin-receptor blockers, angiotensin receptor-neprilysin inhibitor, hydralazine) (1, 74). In patients who are hemodynamically stable with LVOTO, the use of beta-blockers has a negative inotropic effect on basal hypercontractility and reduces the obstruction. In the case of LVOTO, the use of nitrates in order to lower filling pressures may have negative effects by worsening the LVOT gradient and should be avoided (74).

When treating a patient with cardiogenic shock, the approach to therapy is heavily predicated on the presence or absence of LVOT obstruction. In the absence of LVOT obstruction, inotropic agents such as dobutamine or milrinone can be used to augment cardiac output. Vasopressors such as norepinephrine or vasopressin can be utilized as a second-line agent. There have been retrospective studies and case series suggesting the safety and feasibility of using levosimendan (a Ca^2+^ sensitizer) as a potential alternative inotropic agent (75, 76); however, clinical trials are needed to further study this agent before adoption in clinical practice. Several case reports have detailed complex stress CM cases requiring mechanical circulatory support via IABP or percutaneous left ventricular assist devices (such as Impella) until recovery (77, 78).

If LVOTO is present independent of concurrent mitral regurgitation, the use of inotropic support can increase basal hypercontractility and worsen the LVOTO, potentially worsening cardiogenic shock. These patients benefit from measures such as administration of IV fluids and/or the use of low-dose beta-adrenergic antagonists such as metoprolol or esmolol for the purpose of negative inotropy on the hyperdynamic basal myocardium which can reduce the LVOT obstruction. Extreme caution must be practiced with use of beta blockers, however, as despite their benefit in reducing LVOTO they can be potentially detrimental by reducing cardiac output further due to negative inotropy. Other therapeutic agents include vasopressors such as phenylephrine or vasopressin which can be administered to increase systemic vascular resistance and thereby reduce obstruction at the LVOT (1). In refractory cases, ECMO may be used to provide support (79).

Much like with acute management of stress CM, chronic management of stress CM has limited evidence with no official guidelines in place to date. There have been several studies ranging from case series to retrospective studies to meta-analyses investigating various heart failure drugs. Some studies favor ACE-inhibitors and angiotensin receptor blockers over beta blockers in terms of potential long-term mortality benefit while others have shown no significant findings (40, 80, 81). The general approach to chronic care of stress CM patients has been to initiate guideline-directed medical therapy as indicated in the care of the general cardiomyopathy patient. In patients with concurrent coronary artery disease, guideline-directed use of aspirin and statin therapies is likely beneficial as well (74).

### Thromboembolism

In the 2013 ACCF/AHA guidelines for the management of STEMI, there is a Class IIb recommendation to consider prophylactic anticoagulation in patients with anterior wall myocardial infarctions resulting in hypokinetic or akinetic wall motion abnormalities that could pose a potential high risk of LV thrombus formation (82). In a review article by Heckle et al. (66), data compiled from four studies showed a prevalence of 9.2% for thromboembolic events in patients with stress CM. The study posited that an event rate of 9.2% would roughly equate to a score of at least 5 on the CHA2DS2-Vasc scoring system used to determine the need for anticoagulation in patients with atrial fibrillation; therefore, this could theoretically be extrapolated to initiate prophylactic anticoagulation in patients with stress CM (66). To date, there have not been any randomized clinical trials investigating the efficacy of anticoagulation and duration of therapy as a therapeutic or even prophylactic approach to patients with a LV thrombus in the setting of stress CM. Prior retrospective studies have suggested the use of systemic anticoagulation for at least 3 months in patients with a confirmed LV thrombus.

## Prognosis

A retrospective, international, multi-center study by Templin et al., utilized a database called the International Takotsubo Registry to identify risk factors for stress CM as well as and predictors of clinical outcomes in these patients (40). In the study, 21.8% of patients had in-hospital complications including death, which was similar when compared to those with ACS. A multi-variate analysis in this study revealed that physical triggers, acute psychiatric disorders (including anxiety, adjustment disorder, and severe stress), or neurologic disorders (including stroke/TIA, seizures, and headache disorders); a first troponin level >10 times the upper limit of normal; and an LVEF of <45% were all noted to be predictors of higher incidence of in-hospital complications and death. Conversely, older age (>70 years) and the presence of an emotional trigger were associated with a lower incidence of in-hospital complications and death; however, other studies have revealed higher rates of complications in older patients (27, 83).

In-hospital mortality rates for stress CM have ranged between 2 and 5% (32, 83–85). A study in Japan by Isogai et al. (86) compared patients with stress CM due to an out-of-hospital causative factor with those who developed stress CM during hospital admission and found that in-hospital stress CM patients had higher a proportions of co-morbidities along with higher in-hospital mortality (17.9 vs. 5.4%, *p* < 0.001). Presenting in an unstable condition (i.e., post-cardiac arrest or shock requiring pressor support) or having other non-cardiac acute co-morbidities were also associated with a higher risk of in-hospital mortality. In analyzing long-term outcomes, some studies have shown a higher mortality in patients with stress CM compared to a general age and gender-matched population while others have shown no differences in the long term (87, 88).

Data on recurrence have been limited but current evidence reports a recurrence rate of around 2–4% per year and up to as high as 20% in 10 years (40, 88, 89). There are reports of recurrence presenting with a different anatomical variant of stress CM. Some patients may continue to experience symptoms such as fatigue, shortness of breath, chest pain, and exercise intolerance even with recovery of LVEF (1). There is also evidence that patients can experience psychiatric consequences such as post-traumatic stress disorder as a long-term complication of stress CM (90).

## Conclusion

As featured comprehensively in this review, cardiovascular imaging plays a vital role in stress CM and offers many facets of benefits ranging from the initial diagnosis to long-term outcomes. Echocardiography and certain sequences in CMR enable us to differentiate between variants of stress CM, characterize severity of left ventricular dysfunction and identify prognostic features such as LVOTO, mitral regurgitation and RV involvement that often drive our course of therapy. Additionally, several CMR sequences such as feature/tissue tracking, T1/T2 mapping are useful in providing qualitative and quantitative evidence of myocardial injury and degree of inflammation on a cellular and tissue level that can then be translated into prognostic information. As we begin to understand that stress CM is not a benign condition and can often have serious complications in the acute and chronic setting similar to more common cardiac pathologies such as ACS, there is a strong call for further large scale trials in order to investigate and refine our diagnostic and therapeutic approach to this disease.

## Author Contributions

All authors listed have made a substantial, direct, and intellectual contribution to the work and approved it for publication.

## Conflict of Interest

The authors declare that the research was conducted in the absence of any commercial or financial relationships that could be construed as a potential conflict of interest.

## Publisher's Note

All claims expressed in this article are solely those of the authors and do not necessarily represent those of their affiliated organizations, or those of the publisher, the editors and the reviewers. Any product that may be evaluated in this article, or claim that may be made by its manufacturer, is not guaranteed or endorsed by the publisher.
